# PATIENT SATISFACTION WITH REHABILITATION SERVICES FOLLOWING TRAUMATIC BRAIN INJURY: A QUALITY REGISTRY STUDY

**DOI:** 10.2340/jrm.v56.35115

**Published:** 2024-11-13

**Authors:** Camilla G. HOVSET, Cecilie RØE, Helene L. SOBERG, Cathrine BRUNBORG, Eirik HELSETH, Nada ANDELIC, Marit V. FORSLUND

**Affiliations:** 1 Department of Physical Medicine and Rehabilitation, Oslo University Hospital, Oslo; 2 Institute of Clinical Medicine, Faculty of Medicine, University of Oslo, Oslo; 3 Department of Rehabilitation Science and Health Technology, Faculty of Health Sciences, Oslo Metropolitan University, Oslo; 4 Oslo Centre for Biostatistics and Epidemiology, Oslo University Hospital, Oslo; 5 Department of Neurosurgery, Oslo University Hospital, Oslo; 6 Center for Habilitation and Rehabilitation Models and Services (CHARM), Institute of Health and Society, University of Oslo, Norway

**Keywords:** healthcare quality assessment, patient satis-faction, outpatient clinic, primary healthcare, traumatic brain injury

## Abstract

**Objective:**

To examine factors associated with patient satisfaction with rehabilitation services received after traumatic brain injury.

**Design:**

Cross-sectional study.

**Subjects/Patients:**

Persons with mild to severe traumatic brain injury (*n* = 1,375) registered in the “Oslo TBI Registry – Rehabilitation” quality register at Oslo University Hospital from 1 January 2018–31 July 2022.

**Methods:**

Sociodemographics, injury-related variables, patient-reported outcome measures, global functioning, and rehabilitation-related variables were recorded at hospital outpatient visits. The patients reported satisfaction with services received at the outpatient clinic and in primary healthcare at the final follow-up. Multivariable logistic regression models were applied to examine factors associated with patient satisfaction.

**Results:**

Of 316 patients, 83% reported satisfaction with services received at the hospital outpatient clinic. Belief in recovery (odds ratio [OR] = 2.73), shorter time to follow-up (OR = 0.39), and lower symptom burden (OR = 0.96) significantly increased satisfaction. Among 283 patients, 62% reported satisfaction with services in primary healthcare, where belief in recovery (OR = 2.90), shorter time to follow-up (OR = 0.50), higher age (OR = 1.04), and higher number of rehabilitation services received in primary healthcare (OR = 1.32) significantly increased satisfaction.

**Conclusion:**

Across service levels, the strongest associated factors for satisfaction were belief in recovery and shorter time to follow-up, suggesting that timely delivery of traumatic brain injury-related specialized services could increase overall satisfaction.

## LAY ABSTRACT

An increased focus on measuring the quality of healthcare services has led to increased focus on patient experience and evaluation of the services they receive. This study explored how satisfied patients were with the rehabilitation they received after traumatic brain injury, both in a specialized hospital outpatient clinic and in primary healthcare. In total, 83% of patients reported satisfaction with the services in the outpatient clinic, while 62% reported satisfaction with the primary healthcare services. We found that patients’ belief in their recovery and shorter time to follow-up were the 2 strongest factors associated with patient satisfaction across service levels. Lower symptom levels were associated with satisfaction with follow-up at the outpatient clinic, while higher age and high-er number of rehabilitation services received were associated with satisfaction with primary healthcare services. The results indicate that timely specialized traumatic brain injury follow-up could increase overall patient satisfaction.

Every year around 69 million people sustain a traumatic brain injury (TBI) globally, including 1.3 million people in Western Europe and around 16,000 people in Norway (1, 2). While many recover fully, some individuals experience prolonged symptoms for months or years (3) and may be in need of rehabilitation. According to the WHO, rehabilitation can be defined as “a set of interventions designed to optimize functioning and reduce disability in individuals with health conditions in interaction with their environment” (4). The term post-concussion syndrome (PCS) is used for symptoms persisting for more than 3 months (3, 5), and includes physical symptoms such as headache, vertigo, blurred vision, and fatigue; emotional symptoms such as irritation, depression, and anxiety; and cognitive symptoms such as problems with concentration and memory (5, 6). PCS is highly prevalent after mild TBI; The CENTER-TBI study found a prevalence of PCS of 43% in complicated (i.e., verified brain injury on imaging) vs 34% in persons with uncomplicated mild TBI at 6 months post-injury (7). The TRACK-TBI study found that PCS symptoms were common for up to at least a year post-injury, with >50% of persons with mild to severe TBI continuing to affirm 3 or more symptoms at 12 months’ follow-up (8). There is also evidence that persons with moderate-to-severe TBI require long-term follow-ups and rehabilitation services due to cognitive and emotional problems and subsequent disabilities (9).

Individuals with TBI may contact the healthcare system at different times in the recovery process, depending on the injury severity, symptom burden, and availability of services. Recent years have seen an increased focus on measuring healthcare quality and recognition that the patient experience and evaluation of services are important in monitoring and measuring healthcare quality (10, 11).

Within the field of TBI, qualitative research has explored patients’ and family members’ experiences during the rehabilitation process (12–14). A qualitative study from Norway on satisfaction with in-hospital acute care and rehabilitation after severe TBI found that, when asked, 85% of family members were generally satisfied with in-hospital care, treatment, and rehabilitation (13). A Danish study identified that a lack of information concerning rehabilitation options and adapting these to patients’ individual needs after discharge was a common problem (15). Persons with severe TBI received systematic follow-up after hospital discharge, whereas those with milder injuries received varying follow-up through their general practitioner (GP). In Canada, a quantitative study explored the utilization of and satisfaction with community-based health-care after TBI in women only (16). This retrospective cohort study found that women with TBI used more community-based healthcare services and reported that they did not receive the care they needed (especially regarding emotional/mental problems). This study found that there was no significant difference in satisfaction between women with TBI and women without TBI. However, satisfaction with rehabilitation services in the primary healthcare or outpatient specialist healthcare of persons with TBI has not previously been assessed in Norway or any other Scandinavian country.

The aims of the present study were to examine the associations between patient satisfaction with rehabilitation services provided in (*i*) a specialized outpatient clinic and (*ii*) primary healthcare, and sociodemographic data, injury-related data, patient-reported outcome measures (PROMs), global functioning, and use of rehabilitation services.

## METHODS

### Study setting, design and participants

The South-East healthcare region of Norway has a combined population of 3.1 million in 2024 (17). The Oslo University Hospital (OUH) is the only Level 1 trauma centre with neurosurgical services in the South-East region (18), and individuals admitted with TBI from Oslo and adjacent counties are routinely referred for further follow-up at the outpatient TBI clinic at the Department of Physical Medicine and Rehabilitation at OUH. The outpatient TBI clinic is the only specialized outpatient clinic within the public healthcare system in Oslo that provides specialized rehabilitation for those sustaining a TBI. The outpatient clinic has a multidisciplinary team comprising a physician, a physiotherapist, a psychologist/neuropsychologist, an occupational therapist, a social worker, and a team coordinator. Rehabilitation seeks to optimize functioning and employability, and focus on improving physical and mental health, coping, and quality of life. Generally, the patients have their first consultation with a physician for clinical assessment. Depending on the patient’s challenges and needs, the physician may refer the patient to the respective multidisciplinary team members. At the end of the follow-up, there is usually a multidisciplinary summary appointment with the physician and 1 or 2 relevant team members.

Since 2018, the outpatient clinic has registered data from persons with TBI in the “Oslo TBI Registry – Rehabilitation” quality registry. The registry contains information extracted from the electronic patient journal, and quality registry questionnaires with established PROMs and outcomes that the patient and the physician complete separately using pen and paper at the outpatient appointments with the physician. The inclusion criteria for the registry are: (*i*) sustained a TBI of any degree of severity, defined as a Glasgow Coma Scale (GCS) score of 3–15 and an International Classification of Diseases (ICD)-10 diagnosis of TBI with S06.0–S06.9 or T90.5 (if more than 1 year since the injury); (*ii*) age ≥ 18 at inclusion; and (*iii*) resident in Norway. The quality registry has no exclusion criteria. This study presents data from patients registered from 1 January 2018 to 31 July 2022, in a cross-sectional design.

**Fig. 1:**
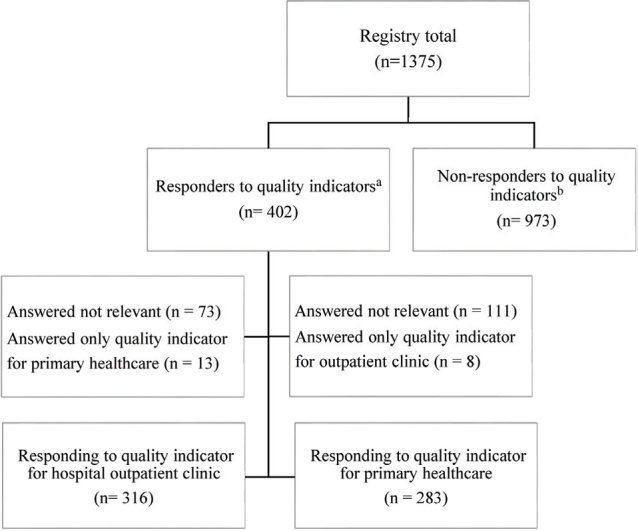
Flowchart of the study population**.**^a^The same patient can answer the quality indicator for both the hospital outpatient clinic and primary healthcare services. ^b^This group also contains patients who were not in need of further treatment/follow-up at the outpatient clinic and therefore not applicable to filling in the quality indicator for the hospital outpatient clinic.

In total, 1,375 unique patients were recorded in the TBI registry during the study period, that is, the registry population. Of these, 402 filled out a quality indicator of satisfaction with rehabilitation services received at the outpatient clinic (*n* = 316) and/or in the primary health services (*n* = 283). Fig. 1 shows a flowchart of the study population.

### Quality indicators

Two quality indicators from the quality registry were the outcome variables of this study: (*i*) “How satisfied are you overall with the rehabilitation/treatment you have received at the outpatient clinic (here) after the injury?”, and (*ii*) “If you have received rehabilitation/treatment in primary healthcare (for example by physiotherapist, occupational therapist, psychologist, general practitioner etc.): How satisfied are you in total with that follow-up?”. The responses were given on a Likert scale with 6 categories (not relevant, not at all, slightly, moderately, fairly, and fully). In the present study, the quality indicators were dichotomized into satisfied (fairly and fully) vs not satisfied (not at all, slightly, and moderately). The patients only completed the quality indicators at the final appointment to evaluate the follow-up they had received. The quality indicators were developed specifically for the quality registry, and have not been formally validated.

### Sociodemographics and injury-related variables

All sociodemographic variables were recorded during follow-up at the outpatient clinic. The variables were gender (male vs female), age in years (continuous), relationship status (married/cohabitant vs single), responsibility for children < 18 years (yes vs no), education level (≤ 12 years vs > 12 years), and employment status (employed [full or part-time work/student] vs unemployed [full sick leave, receiving disability benefits or work clearance allowance, retired, homemaker]).

The injury-related variables were acute GCS score (continuous) (19), intracranial injury verified on CT or MRI imaging (yes vs no), and time to follow-up at the outpatient clinic since injury in months (continuous). Information concerning the GCS score originated from hospital records or the patient, and was recorded as a number ranging from 3 to 15. The TBI severity was classified according to the GCS score as mild (GCS 13–15), moderate (GCS 9–12), or severe (GCS 3–8). Intracranial injury was determined from the ICD-10 diagnosis, where S06.1–S06.9 was categorized as intracranial injury, and S06.0 as no intracranial injury. These variables were recorded by the physician in the quality registry questionnaire during follow-ups at the outpatient clinic.

### Patient-reported outcome measures

The Rivermead Post-concussion symptoms Questionnaire (RPQ) assessed the level of symptom burden after TBI. The RPQ is a 16-item questionnaire with a total score range from 0 to 64 (best to worst) (20). Scores of 1 (no longer a problem) were rescored as 0 as recommended by King *et al*. (20). A total score of >16 is considered clinically relevant (21).

Depressive symptoms were assessed with the Patient Health Questionnaire (PHQ-9), a 9-item questionnaire with a total score range from 0 to 27 (best to worst) (22). A total score of ≥10 is considered a clinically relevant level of depressive symptoms (23).

Anxiety symptoms were assessed with the Generalized Anxiety Disorder (GAD-7), a 7-item questionnaire with a total score range from 0 to 21 (best to worst) (24). The clinically relevant cut-off for anxiety can be set at ≥8 (25).

Post-traumatic stress symptoms were assessed using the Post Traumatic Symptom Scale-10 (PTSS-10), a 10-item questionnaire with a range from 10 to 70 (best to worst) (26, 27). A cut-off of ≥35 is considered clinically relevant (27).

Belief in recovery was assessed through the quality registry question: “Do you have substantial belief in recovery after your injury?” The variable was dichotomized into yes (responses “yes” and “I do not have any symptoms anymore”) vs no (“no” and “I don’t know”). The wording in Norwegian regarding “recovery” may be interpreted as recovery ranging from some improvement to full recovery. The single question on belief in recovery was developed specifically for the quality registry and has not been formally validated.

### Global functioning

Global functioning was measured with the Glasgow Outcome Scale-Extended (GOSE) (28). The GOSE has the following outcome categories: 1 = dead, 2 = vegetative state, 3 = lower severe disability, 4 = upper severe disability, 5 = lower moderate disability, 6 = upper moderate disability, 7 = lower good recovery, and 8 = upper good recovery (implying full functional recovery). In the present study, the patients were dichotomized into moderate to severe disability (GOSE 3–6) versus good recovery (GOSE 7–8).

### Rehabilitation services

The variable multidisciplinary team (no vs yes) divides those who only received follow-up by the physician from those who received additional follow-up by other multidisciplinary team members at the hospital outpatient clinic. This was recorded by the physician in the quality registry questionnaire at follow-ups at the outpatient clinic.

The number of primary healthcare services summarizes the number of patient self-reported rehabilitation services received from a GP, physiotherapist, occupational therapist, chiropractor, psychologist, optician, and vocational support/follow-up from the Norwegian Labour and Welfare Organization in the community (NAV). This was recorded by the patient in the quality registry questionnaire at follow-ups at the outpatient clinic.

### Statistical analysis

The statistical analyses were conducted using IBM SPSS version 29 (IBM Corp, Armonk, NY, USA). The significance level was set at *p* < 0.05. Descriptive statistics were used to describe the registry population and the subpopulation of quality indicator responders compared with non-responders, using data from the first follow-up at the outpatient clinic. An independent sample *t*-test was used for age (normal distribution) and the Mann–Whitney *U* test for the rest of the continuous variables (skewed distribution), whereas the χ^2^ test was used for categorical variables to compare responders with non-responders to the quality indicators. Missing values in the PROMs (RPQ, PHQ-9, GAD-7 and PTSS-10) were handled by imputation for those missing 1 or 2 items by using the mean item score, calculated by dividing the sum scores of items by the number of items answered. Participants missing more than 2 items were not included in the analysis. Time to follow-up was ln-transformed and corrected for outliers by truncation (with outliers >60 months recoded as 60 months).

Binary logistic regression was performed to examine factors associated with the quality indicators, using data from the final follow-up at the outpatient clinic (for the responding group). Factors were included in the multivariable models based mainly on knowledge from the literature and expert opinion (29–32), but also using information from univariate analysis on variables with *p*-values of < 0.2. The following factors were included in the full models: age, gender, education level, intracranial injury, time to follow-up, continuous GCS score, GOSE, belief in recovery, RPQ, and rehabilitation services (multidisciplinary team for the outpatient clinic and number of services received in primary healthcare for the primary healthcare quality indicator). Variables were examined for normality, outliers, and multicollinearity. The RPQ, PHQ-9, GAD-7, and PTSS-10 as continuous variables were all highly correlated at >0.7; consequently, only the RPQ was used in the models as the RPQ captures physical, cognitive, and emotional aspects after TBI. We present the full model for each quality indicator and the best-fit model after the subsequent backward elimination of factors. The results are presented with odds ratio (OR), a 95% confidence interval (CI), and *p*-value. An OR > 1 increases the probability of patient satisfaction, and OR < 1 decreases the probability of patient satisfaction with services.

Due to 6.1% missing data in the full model for the hospital outpatient clinic and 7.1% missing data for primary healthcare, a sensitivity analysis was conducted generating 10 multiple imputed data sets. These analyses showed similar results to the full models without imputation (data not shown); therefore, we retained the full models without imputation.

The predictive accuracy of the model was assessed through calibration using the Hosmer–Lemeshow goodness-of-fit test. A non-significant Hosmer–Lemeshow goodness-of-fit test (*p*>0.05) indicates satisfactory fit. Discrimination of the models was assessed using the area under the receiver operating characteristic curve (AUC-ROC). An AUC-ROC score of >0.7 indicated an acceptable discriminatory ability of the model.

## RESULTS

Table I describes the registry population and subpopulations of quality indicator responders and non-responders. For the registry population (*n* = 1,375), 52% were women, the mean age was 42.2 (SD 14.4) and 91.0% had a mild TBI. When comparing the group of responders with the group of non-responders with the quality indicators, the responders group had significantly more women, a higher number of married/cohabitating individuals, and a higher education level. Furthermore, the responders group had a slightly longer time to follow-up, fewer individuals with violence as cause of injury, and a slightly lower percentage of individuals with an intracranial injury. The responders reported higher symptom burdens related to brain injury (RPQ), more post-traumatic symptoms (PTSS-10), and the group had a higher percentage of individuals with severe to moderate disability (GOSE). Age, work status, GCS, PHQ-9, GAD-7, and belief in recovery did not differ between the 2 groups.

**Table I:** Characteristics of the registry population and differences between responders and non-responders to the quality indicators

Variable	Registry total, *n* = 1,375	Responders quality indicators^a^ *n* = 402	Non-responders quality indicators *n* = 973	Comparison of responders vs non-responders *p*-value
*Sociodemographics*				
Gender				0.019*
Male	661 (48.1)	173 (43.0)	488 (50.2)	
Female	714 (51.9)	229 (57.0)	485 (49.8)	
Age (in years), mean (SD)	42.2 (14.4)	42.4 (13.5)	42.1 (14.8)	0.714
Marital status				0.019*
Single	614 (44.7)	160 (39.8)	455 (46.8)	
Married/cohabitating	755 (55.1)	242 (60.2)	515 (52.9)	
Missing	3 (0.2)	0	3 (0.3)	
Level of education				< 0.001*
Lower education ≤ 12 years	402 (29.2)	90 (22.4)	312 (32.1)	
Higher education ≥ 13 years	947 (68.9)	309 (76.9)	638 (65.6)	
Missing	26 (1.9)	3 (0.7)	23 (2.4)	
Work status				0.743
Not working	715 (52.0)	207 (51.5)	508 (52.2)	
Working	652 (47.7)	195 (48.5)	457 (47.0)	
Missing	8 (0.6)	0	8 (0.8)	
*Injury-related variables*				
Time to follow-up^b^, median (IQR)	3.0 (2-6)	4.0 (2-8)	3.0 (1-5)	< 0.001*
GCS, mean (SD)	14.1 (2.3)	14.2 (2.2)	14.1 (2.3)	0.350
Mild (GCS 13–15)	1251 (91.0)	370 (92.0)	881 (90.5)	
Moderate (GCS 9–12)	56 (4.1)	12 (3.0)	44 (4.5)	
Severe (GCS 13–15)	67 (4.9)	20 (5.0)	42 (4.8)	
Missing	20 (1.5)	5 (1.3)	15 (1.5)	
Cause of injury				0.028*
Fall	671 (48.8)	195 (48.5)	476 (48.9)	
Traffic	304 (22.1)	93 (23.1)	211 (21.7)	
Violence	103 (7.5)	18 (4.5)	85 (8.7)	
Other	285 (20.7)	94 (23.4)	191 (19.6)	
Missing	12 (0.9)	2 (0.5)	10 (0.1)	
Intracranial injury				0.028*
No	916 (66.6)	286 (71.1)	630 (64.7)	
Yes	458 (33.3)	116 (28.9)	342 (35.1)	
Missing	1 (0.1)	0	1 (0.1)	
*Patient-reported outcome measures*				
RPQ, mean (SD)	23.6 (15.7)	25.3 (15.0)	22.8 (15.9)	0.008*
PHQ-9, mean (SD)	8.9 (6.2)	9.2 (5.7)	8.8 (6.4)	0.103
GAD-7, mean (SD)	5.7 (5.2)	5.7 (4.8)	5.7 (5.3)	0.372
PTSS-10, mean (SD)	25.7 (13.5)	26.7 (12.9)	25.3 (13.7)	0.010*
Belief in recovery				0.982
No	265 (19.3)	82 (20.4)	183 (18.8)	
Yes	964 (70.1)	299 (74.4)	665 (68.3)	
Missing	146 (10.6)	21 (5.2)	125 (12.8)	
*Global functioning*				
GOSE^c^				0.006*
GOSE 3–6	1,079 (78.5)	336 (83.6)	743 (76.4)	
GOSE 7–8	290 (21.1)	63 (15.7)	227 (23.3)	
Missing	6 (0.4)	3 (0.7)	3 (0.3)	

a Responders to the quality indicator on the outpatient clinic services and the primary healthcare services were assessed together as 1 population.

b Time in months from injury until first registered consultation, outliers >60 months recoded as 60 (not ln transformed).

c GOSE 3–6 = severe to moderate disability and GOSE 7–8 = good recovery.

GCS: Glasgow Coma Scale; RPQ: Rivermead Post-concussion symptom Questionnaire; PHQ-9: Patient Health Questionnaire-9; GAD-7: General anxiety disorder-7; PTSS-10: Post Traumatic Symptom Scale; GOSE: Glasgow Outcome Scale Extended.

* Significant values *p* < 0.05.

The percentage of patients who received treatment or follow-up by the multidisciplinary team was significantly higher in the responding group (72.6%) compared with the non-responding group (51.1%) (*p* < 0.001), whereas the remaining patients received follow-up only by the physician.

The median number of received primary healthcare services was 1.0 (range 0–6, IQR 0–2) in the total population and 1.0 (range 0–6, IQR 0–2) in both the responding and the non-responding group. In the responding group, most patients reported that they have received follow-up by their GP (61.9%), followed by physiotherapist/chiropractor (60.9%), occupational therapist (24.9%), psychologist (22.9%), optician (15.4%), and vocational/follow-up from NAV (11.4%).

**Fig. 2:**
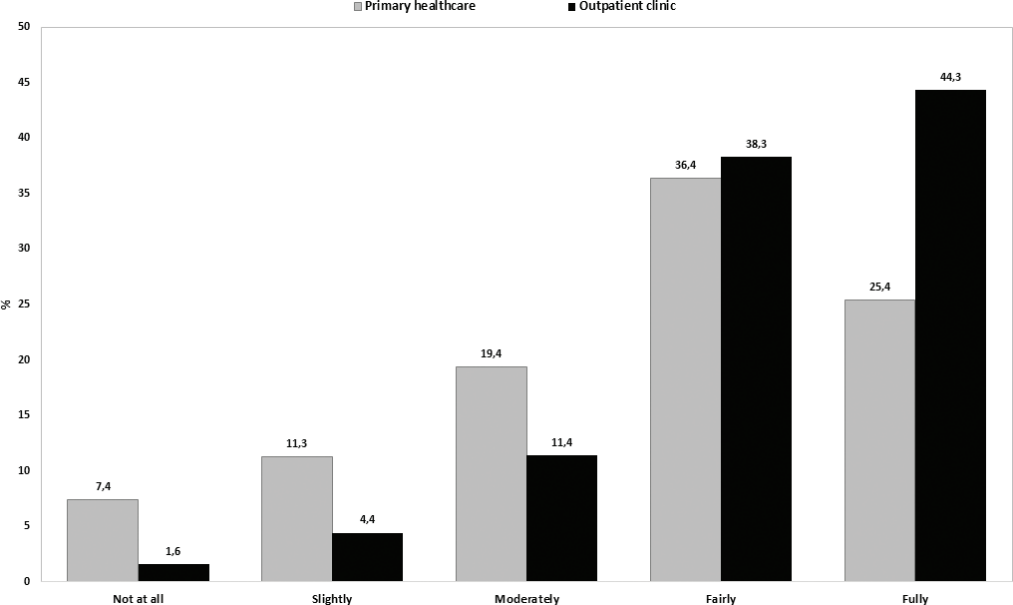
Frequency distribution (%) of responses on patient satisfaction with services in primary healthcare (*n* = 283) and at the hospital outpatient clinic (*n* = 316).

Fig. 2 shows the distribution of responses to the quality indicators. For patient satisfaction with the outpatient services, the responses were highly skewed towards patients reporting satisfaction, with the most frequent response being “fully satisfied” (44.3%). Regarding the satisfaction with primary healthcare services, the responses were more spread out over all alternatives. The most frequent response was “fairly satisfied” (36.4%). A higher frequency of responders reported being “not at all satisfied” with the primary healthcare services (7.4%) as compared with the outpatient services (1.6%).

### Factors indicating satisfaction with the services in the hospital outpatient traumatic brain injury clinic

Most patients (82.6%) reported that they were satisfied with the services at the outpatient clinic. The multivariable regression showed that belief in recovery was significantly associated with increased odds of being satisfied, whereas longer time to follow-up and higher symptom burden (RPQ) decreased the probability of a favourable satisfaction outcome (Table II). A higher age tended to increase the odds of being satisfied, and being male tended to decrease the odds, although these factors were not significantly associated with the outcome.

**Table II:** Multivariable logistic regression model for patient satisfaction with the services in the hospital outpatient traumatic brain injury clinic (*n* = 316)

Variable	Full model OR (95% CI)	*p*-value	Best fit-model OR (95% CI)	*p*-value
Age in years	1.03 (0.997–1.056)	0.080	1.02 (0.996–1.053)	0.090
Gender (0 = female, 1 = male)	0.47 (0.220–1.015)	0.055	0.52 (0.246–1.091)	0.083
Education (0 = ≤12 years, 1 = ≥13 years)	0.64 (0.255–1.616)	0.347		
Intracranial injury (0 = no, 1 = yes)	1.17 (0.369–3.691)	0.793		
Time to follow-up in months	0.40 (0.222–0.734)	0.003*	0.39 (0.218–0.708)	0.002*
GCS score	1.13 (0.936–1.339)	0.186	1.10 (0.950–1.283)	0.199
GOSE (0 = GOSE 3–6, 1 = GOSE 7–8)	1.69 (0.706–4.046)	0.238	1.68 (0.697–4.025)	0.249
RPQ total score	0.96 (0.936–0.990)	0.007*	0.96 (0.938–0.990)	0.008*
Belief in recovery (0 = no, 1 = yes)	2.67 (1.280–5.581)	0.009*	2.73 (1.314–5.681)	0.007*
Multidisciplinary team (0 = physician only, 1 = multidisciplinary team)	0.48 (0.148–1.579)	0.229	0.47 (0.149–1.465)	0.192

An OR >1 increases the likelihood (odds) of patient satisfaction with the services, OR < 1 reduces the likelihood.

GCS: Glasgow Coma Scale; GOSE: Glasgow Outcome Scale Extended; RPQ: Rivermead Post-concussion symptoms Questionnaire.

* Significant values *p* < 0.05.

The Hosmer–Lemeshow goodness-of-fit test demonstrated good fit in both the full model (*p* = 0.320) and the best-fit model (*p* = 0.133). The AUC-ROC was 0.817 for the full model and 0.815 for the best-fit model, indicating acceptable discriminatory ability for both models.

### Factors indicating satisfaction with services in primary healthcare

More than half (61.8%) of the patients were satisfied with rehabilitation/follow-up in primary healthcare. The multivariable regression showed that belief in recovery, a higher number of services received in primary healthcare, and a higher age increased the odds of a favourable patient satisfaction outcome, whereas a longer time to follow-up decreased the odds (see Table III).

**Table III:** Multivariable logistic regression model for satisfaction with the rehabilitation/treatment in primary healthcare services (*n* = 283)

Variable	Full model OR (95% CI)	*p*-value	Best-fit model OR (95% CI)	*p*-value
Age in years	1.04 (1.014–1.064)	0.002*	1.04 (1.015–1.062)	0.001*
Gender (0 = female, 1 = male)	0.86 (0.464–1.589)	0.627		
Education (0 = ≤12 years, 1 = ≥13 years)	1.20 (0.568–2.538)	0.631		
Intracranial injury (0 = no, 1 = yes)	0.82 (0.324–2.058)	0.667		
Time to follow-up in months	0.47 (0.288–0.756)	0.002*	0.50 (0.315–0.781)	0.002*
GCS score	0.83 (0.659–1.035)	0.097	0.87 (0.722–1.045)	0.136
GOSE (0 = GOSE 3–6, 1 = GOSE 7–8)	1.84 (0.905–3.751)	0.092	1.80 (0.942–3.447)	0.075
RPQ total score	1.00 (0.975–1.020)	0.789		
Belief in recovery (0 = no, 1 = yes)	2.73 (1.410–5.290)	0.003*	2.90 (1.554–5.394)	< 0.001*
Number of rehabilitation services	1.34 (1.079–1.662)	0.008*	1.32 (1.067–1.626)	0.010*

An OR >1 increases the likelihood (odds) of satisfaction with the treatment, OR < 1 reduces the likelihood.

GCS: Glasgow Coma Scale; GOSE: Glasgow Outcome Scale Extended; RPQ: Rivermead Post-concussion symptoms Questionnaire.

* Significant values *p* < 0.05.

The Hosmer–Lemeshow goodness-of-fit test demonstrated good fit in both the full model (*p* = 0.151) and the best-fit model (*p* = 0.529). The AUC-ROC values were 0.747 for the full model and 0.746 for the best-fit model, indicating that these models also had acceptable discriminatory ability.

## DISCUSSION

This study assessed patients’ satisfaction with the rehabilitation services received at a hospital outpatient clinic and in primary healthcare following TBI, and explored the association between patient satisfaction and different patient characteristics, PROMs, global functioning, and injury-related and rehabilitation data. Generally, patients were satisfied with the follow-up they received; 83% were satisfied with the outpatient clinic and 62% with the primary healthcare services. The high percentage of satisfaction with outpatient services could be explained by the individualized follow-up provided by the multidisciplinary team with specialized TBI competence. The knowledge and experience regarding TBI may be more variable among care providers in primary healthcare services and could explain the difference in satisfaction between the outpatient clinic and primary healthcare. The difference could also be due to the timing of the quality indicators, which were filled in at a time where patients had just received services at the outpatient clinic, while services received in primary healthcare might be further back in time (33).

The strongest factor associated with patient satisfaction overall was belief in recovery. Patients who believed in recovery had 2.73 (outpatient clinic) and 2.90 (primary healthcare) times higher odds of being satisfied with the services they received. A systematic review of the relationship between patients’ recovery expectations and health outcomes (34) found that positive expectations were associated with better health outcomes in 15 of 16 studies. Further, a study by Snell et al. (29) assessed 147 persons with mild TBI and found that patients’ beliefs regarding their injury and recovery had significant associations with outcome over time. Patients may experience conflict between their expected and actual recovery over time and therefore perceive the injury as more serious than they did initially, which may subsequently influence their belief in recovery. Thus, it is important to target the beliefs concerning the recovery course and prognosis in the early stages of injury to avoid them becoming fixed and less malleable with time (29).

For both the hospital outpatient clinic and primary healthcare, longer time from injury to follow-up was associated with decreased odds of being satisfied. For each month longer the patient had to wait for follow-up, the odds of patient satisfaction decreased 61% for the outpatient clinic services and 50% for primary healthcare services. This shows that the time from injury until follow-up at the specialized rehabilitation outpatient clinic was important to patients. Possible explanations for this association could be that patients feel more secure regarding the type of services provided in primary healthcare after receiving information and guidance from specialized healthcare, a synergistic effect on satisfaction when receiving specialized healthcare alongside primary healthcare services, or the finding may serve as a proxy for collaboration between healthcare service levels. A qualitative study by Déry *et al*. (12) explored how individuals with mild TBI experienced the waiting time from referral to receiving specialized interdisciplinary rehabilitation services. The perception depended on their experience with other health services received during that time. The patients experienced uncertainty due to lack of information regarding recovery and availability of health services, which exacerbated their symptoms. Many felt that if they had received timely care, the services would have helped them to manage symptoms and avoid chronification.

The present study found that for the outpatient clinic higher symptom burden (RPQ) was associated with lower odds of being satisfied with the rehabilitation services. Patients with more post-concussion symptoms may have other expectations regarding treatment and symptom relief, which might not be met. Self-perceived unmet healthcare needs have been shown to negatively affect patient satisfaction with care among Canadians living with neurological conditions (30). The association could also be related to other factors not explored here, such as comorbidity and self-rated health, which might affect both symptom burden and satisfaction (32, 35).

In the current study, we found that a higher age increased the odds of being satisfied with the services received in primary healthcare, although not significantly for the outpatient clinic. A Norwegian study of 225 patients on satisfaction with services received at the emergency ward (after-hours care in primary healthcare) (31) similarly found that age was associated with satisfaction. Older patients were more likely to be satisfied with the overall services received at the emergency ward. They might have lower expectations and different attitudes towards treatment and recovery than younger patients. Differences in life stages could contribute to this, as some younger patients might experience a larger gap between function and demand, for example if they care for young children or have less job stability and experience.

Increasing the number of services received in primary healthcare increased the probability of patients being satisfied with the services received. This was also found by Pound *et al*. (36) in a study on satisfaction with stroke care, where patients with more therapy (measured in units of physiotherapy, speech therapy, and occupational therapy received at home) reported increased satisfaction (up to a certain point). A possible reason for increased satisfaction with higher number of services could be that the patients who received more services experience fewer unmet healthcare needs.

In our study, we found a tendency for males to be less satisfied with outpatient rehabilitation services. Previous research shows inconsistency when reporting satisfaction with healthcare among men and women in general; studies have found that women can be both less and more satisfied with treatment than men (36, 37). Several studies show that men are more likely to sustain a TBI and are overrepresented in the TBI population, including the mild TBI population, as reported in a study by CENTER-TBI (64% men) (38). In this study sample, women were more numerous in the group of quality indicator responders (57%). Women are known to seek more help for their symptoms (39), which might partly explain why more women are in the responding group. Further, women are more likely to experience more severe post-concussion symptoms than men (38). This holds true for the present sample where women reported a mean RPQ total score of 29.8 as compared with 19.4 among the men in the responding group, and individuals with higher symptom burdens were more likely to be referred for further follow-up by the multidisciplinary team. A much larger percentage of men in the responding group had an intracranial injury compared with the women (45.7% vs 16.2%), and they were more likely to be referred directly from the Neurosurgical Department to the hospital outpatient clinic for follow-ups and driving licence assessments. According to Norwegian regulations, all those with TBI with a verified intracranial injury receive a driving ban lasting a minimum of 6 months after injury and require specialized assessment before the ban can be lifted. We can only speculate that one reason for lower odds of satisfaction among men comes from driving restrictions.

The greatest study limitation was the low response rate to the quality indicators. Approximately 70% of patients in the registry population were referred for further follow-up at the outpatient clinic; therefore, they should have completed the quality indicators at the last appointment. However, only around 30% of patients were registered with quality indicator data. The exact number of patients lost to follow-up or who were not referred to follow-up is not known, as it was not possible to compare the hospital administrative patient registry with those included in our quality registry. A lack of data in the registry could be due to patient preferences, follow-up dropouts, a lack of distribution of the questionnaires, or lack of registering/plotting the data in the registry. We know that some data were lost due to the COVID-19 pandemic, when consultations were conducted by telephone for several months, and patients could not complete the questionnaires. Variability has occurred in physician adherence to the quality registry procedure, although we have no reason to suspect systematic selection of responders or non-responders. The total registry population and the responding group seem to be comparable, although some differences are expected based on the selection of patients referred to the multidisciplinary team due to higher symptom burden and lower global functioning. Based on the population characteristics, the findings would be generalizable to predominantly mild TBI populations. Other important limitations are the use of single questions for assessing patient satisfaction and belief in recovery instead of validated tools. This was done to reduce the length of the registry questionnaires and patient burden in a clinical consultation.

This study does not explore what elements of the services received the patients are satisfied with, or how the service quality can be improved. A qualitative study will be conducted in the next step of this project to further explore patients’ experience and satisfaction with rehabilitation services.

In sum, 83% of patients reported satisfaction with services received at the hospital outpatient clinic, where belief in recovery, shorter time to follow-up, and lower symptom burden significantly increased the odds of satisfaction. More than half (62%) reported satisfaction with services in primary healthcare, where belief in recovery, shorter time to follow-up, higher age, and higher number of rehabilitation services received in primary healthcare significantly increased the satisfaction odds. In conclusion, the findings highlight the importance of patient belief in recovery, and suggest that timely delivery of specialized follow-up with information on the recovery process and prognosis after TBI could increase overall patient satisfaction.
